# Apolipoprotein C-II Deposition Amyloidosis: A Potential Misdiagnosis as Light Chain Amyloidosis

**DOI:** 10.1155/2016/8690642

**Published:** 2016-10-20

**Authors:** Sadichhya Lohani, Emily Schuiteman, Lohit Garg, Dhiraj Yadav, Sami Zarouk

**Affiliations:** ^1^Department of Internal Medicine, Beaumont Health, Royal Oak, MI 48073, USA; ^2^Lehigh Valley Health Network, Allentown, PA 18103, USA; ^3^University Hospital Seidman Cancer Center, Case Western University, Cleveland, OH 44106, USA; ^4^Department of Nephrology, Beaumont Health, Royal Oak, MI 48073, USA

## Abstract

Hereditary amyloidoses are rare and pose a diagnostic challenge. We report a case of hereditary amyloidosis associated with apolipoprotein C-II deposition in a 61-year-old female presenting with renal failure and nephrotic syndrome misdiagnosed as light chain amyloidosis. Renal biopsy was consistent with amyloidosis on microscopy; however, immunofluorescence was inconclusive for the type of amyloid protein. Monoclonal gammopathy evaluation revealed kappa light chain. Bone marrow biopsy revealed minimal involvement with amyloidosis with kappa monotypic plasma cells on flow cytometry. She was started on chemotherapy for light chain amyloidosis. She was referred to the Mayo clinic where laser microdissection and liquid chromatography mass spectrometry detected high levels of apolipoprotein C-II, making a definitive diagnosis. Apolipoprotein C-II is a component of very low-density lipoprotein and aggregates in lipid-free conditions to form amyloid fibrils. The identification of apolipoprotein C-II as the cause of amyloidosis cannot be solely made with routine microscopy or immunofluorescence. Further evaluation of biopsy specimens with laser microdissection and mass spectrometry and DNA sequencing of exons should be done routinely in patients with amyloidoses for definitive diagnosis. Our case highlights the importance of determining the subtype of amyloidosis that is critical for avoiding unnecessary therapy such as chemotherapy.

## 1. Introduction

Amyloidosis comprises a group of diseases caused by the extracellular deposition of toxic insoluble polymeric fibrillar proteins in tissues and organs, resulting in organ dysfunction [1]. Misfolded proteins aggregate in a characteristic *β*-pleated sheet configuration that allows them to bind to Congo red dye, producing apple green birefringence under polarized light [1]. Light chain amyloidosis (AL) is the most common cause of systemic amyloidoses in North America [1]. Hereditary or familial amyloidoses are autosomal dominant diseases [2] which are caused by an inherited mutation of protein with amyloidogenic potential [1–3]; they are a rare form of systemic amyloidoses and have an incidence of <1 per 100,000. Hereditary amyloidoses are difficult to be correctly identified and thus are frequently misdiagnosed and treated inappropriately [4]. Transthyretin (TTR), lysozyme, gelsolin, cystatin C, fibrinogen A*α*, and apolipoproteins A-I or A-II are the commonly described proteins associated with hereditary amyloidoses, of which familial transthyretin-associated amyloidosis (ATTR) is the most common [1, 4–6].

Apolipoprotein C-II has been found to have amyloidogenic potential in numerous in vitro studies [7]. Human apolipoprotein C-II is one of the several lipid-binding proteins that can self-aggregate in favorable conditions, forming fibrils that accumulate in tissues to create amyloid deposits [6]. The kidneys are a very common site for amyloid deposition in most forms of systemic amyloidosis because of the favorable extracellular environment for amyloid formation and stabilization [1]. Renal amyloidosis usually manifests as nephrotic range proteinuria and acute kidney injury that progresses to end stage renal disease without treatment [1].

In general, the diagnosis of amyloidosis requires identification of amyloid in the abdominal fat or affected tissue through biopsy; apple green birefringence on polarized light and randomly arranged fibrillar proteins on electron microscope are characteristic. However, identification of the type of amyloid protein is important for accurate diagnosis and appropriate treatment of the particular type of amyloidosis [8]. Laser microdissection and mass spectrometry (LMD/MS) is one such highly sensitive and specific method [8]. Our case highlights the clinical presentation of apolipoprotein C-II renal amyloidosis as nephrotic syndrome, a diagnostic challenge with potential misdiagnosis as light chain amyloidosis and the importance of LMD/MS technique used to make the definitive diagnosis of apolipoprotein C-II associated amyloidosis.

## 2. Case Presentation

A 61-year-old Caucasian female with no past history of renal disease presented to the office with uncontrolled hypertension, averaging 160/90 mmHg, and gradually worsening bilateral leg swelling for one year. Review of systems was positive for morning periorbital edema, dyspnea on exertion, fatigue, and twenty-pound weight loss in six months. She did not have any dietary restrictions and was not using any nonsteroidal anti-inflammatory medications (NSAIDs). She was being managed with multiple antihypertensive drugs over the past year. Her medications included furosemide 20 mg once daily and hydralazine 25 mg three times a day. She was previously on lisinopril, losartan-hydrochlorothiazide, and amlodipine, all of which were eventually discontinued due to adverse effects.

Her past medical history consisted of hypertension for three years, hypothyroidism, depression, anxiety, benign colonic polyps, mitral valve prolapse, and hyperlipidemia. Surgical history included cholecystectomy, tonsillectomy, and adenoidectomy. She had a family history of coronary artery disease, hypertension in her father, and breast and uterine cancer in her sister. She had an 18-pack-year history of smoking but had quit 29 years ago. She would drink only on social occasions and had no history of recreational drug use. In addition to her antihypertensive medications, her other medications included levothyroxine, alprazolam, escitalopram, and atorvastatin.

On physical examination, her blood pressure was 190/100 mmHg. She had bilateral pitting pedal edema. Respiratory, cardiovascular, and abdominal examinations were unremarkable. She had a creatinine of 2.3 mg/dL that was elevated from her baseline creatinine of 1.3 mg/dL one year ago. Complete blood counts, electrolytes, and liver function panel were normal as illustrated in Table 1. She was noted to have persistent >3+ proteinuria on dipstick for the last 3 years prior to the presentation. At the time of presentation, urinalysis redemonstrated >3+ proteinuria and 2+ blood with red blood cells of 11–24 per high power field. Urine protein-to-creatinine ratio was elevated at 6.3 mg/g (<0.2 mg/g). Albumin was low 3.4 mg/dL (Table 1). Infectious and autoimmune workup was negative (Table 1).

Chest X-ray showed bilateral pleural effusions. Stress myocardial perfusion imaging revealed normal ventricular wall thickness and normal systolic ejection fraction. Serum protein electrophoresis with immunofixation showed immunoglobulin A (IgA) kappa monoclonal protein present as two bands in the beta globulin region. Free kappa light chain level was 2.45 (0.35–2.49 mg/dL) and free lambda level was 0.93 (0.5–2.71 mg/dL), with ratio elevated at 3.91 (0.27–1.8).

**Figure 1:**
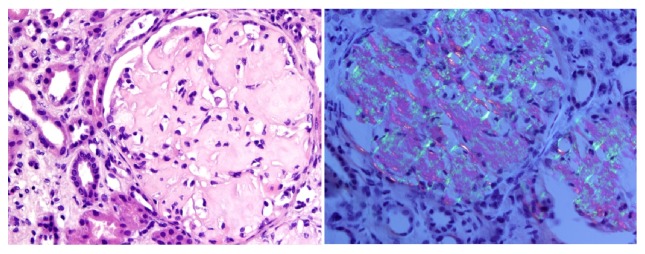
H&E (100x and 500x magnification) and Congo red staining/Polarized Congo red. Showing multiple glomeruli with an expansion of the mesangial matrix by amorphous, pale eosinophilic, acellular material and orange-red staining of eosinophilic material with apple green birefringence on polarization microscopy.

**Figure 2:**
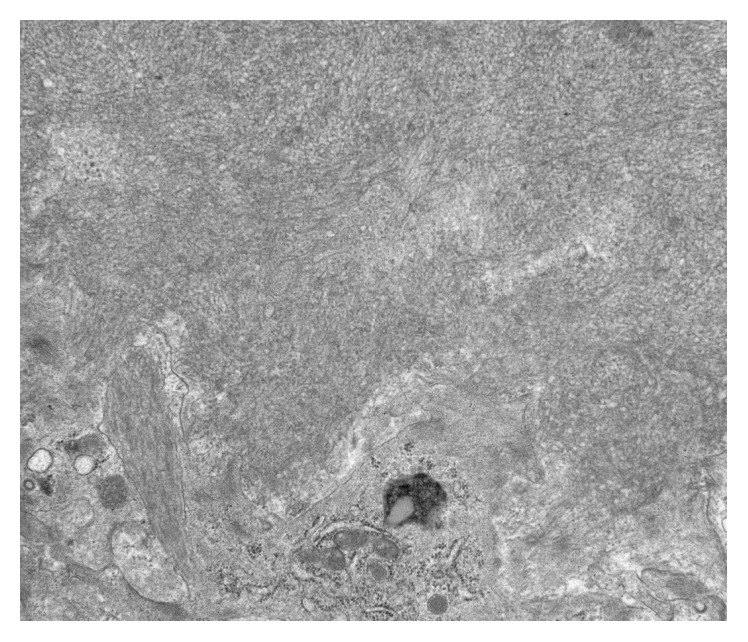
Electron microscopy. Showing deposition of small, randomly arranged fibrils in the mesangium with features characteristic of amyloid fibrils.

The patient was started on losartan and spironolactone, and the dose of her hydralazine and furosemide was increased. After her blood pressure was controlled, she underwent a renal biopsy one month later. The renal biopsy revealed amorphous, pale eosinophilic, acellular material in the mesangium, tubular wall, and walls of arterioles. The eosinophilic material exhibited apple green birefringence on polarization microscopy after Congo red staining. These findings were consistent with amyloidosis (Figure 1). Electron microscopy showed randomly arranged fibrils in the mesangium with features characteristic of amyloid fibrils (Figure 2). Immunofluorescence reactions with antibodies against IgG, IgA, IgM, C1q, and kappa and lambda light chains were negative in the glomeruli. A positive reaction was seen with C3 in the glomeruli. Subsequently, bone marrow biopsy was done which showed 6% plasmacytosis and minimal involvement with amyloidosis. Flow cytometry revealed kappa monotypic plasma cells in the bone marrow. A bone survey was negative for lytic lesions and positron emission tomography (PET) scan did not reveal increased uptake to suggest malignancy.

With a presumptive diagnosis of kappa light chain amyloidosis based on the bone marrow biopsy findings and renal amyloid deposition, she was started on combination chemotherapy with cyclophosphamide, bortezomib, and dexamethasone for presumed AL amyloidosis. During this time, evaluation of kidney tissue was done at the Mayo clinic with laser microdissection (LMD) and liquid chromatography mass spectrometry (LCMS) on peptides extracted from Congo red positive dissected areas. LCMS detected high levels of apolipoprotein C-II. Analysis for lambda or kappa light chains, transthyretin, and serum amyloid A was negative. It was concluded that these findings are unequivocally consistent with apolipoprotein C-II amyloid involving the mesangium.

Chemotherapy was stopped after the mass spectrometry results were available; in total, the patient only completed two short courses. Genetic testing was performed by direct sequencing of apolipoprotein C-II and detected a mutation at codon 69 with glutamate to valine substitution due to heterozygous c.206A→T transition in exon 3. This missense mutation was detected in the patient as well as her son, but not her daughter.

## 3. Discussion

Amyloid is basically fragments of protein that are misfolded by various mechanisms, lose their normal function, and aggregate to form deposits in the extracellular space [1, 5–9]. There are more than 20 structurally different polypeptides and proteins known to cause amyloidosis in vivo [10]. The biochemical nature of these proteins in the fibril deposits defines the individual amyloid diseases and determines their diagnosis and treatment [1–8]. Because hereditary amyloidoses are so rare, they are not usually considered in the differential diagnosis of systemic amyloidoses in absence of family history [4, 5]. However, due to their variable penetrance [4–7] they can sometimes first manifest sporadically, as is seen in our case.

The kidney is one of the most frequently involved organs in amyloidosis, including apolipoprotein associated hereditary amyloidosis [5]. Renal amyloidosis usually manifests as proteinuria [11] often resulting in nephrotic syndrome. This is associated with hypoalbuminemia, hypercholesterolemia, edema, and anasarca [5]. Nonspecific symptoms such as generalized fatigue and anorexia in the presence of symptoms pertinent to particular organ dysfunction should prompt initial workup [1]. Clinical presentation for hereditary amyloidoses, including apolipoprotein C-II associated disease, is similar to those of patients with AL. Thus, unless the protein is identified and in the absence of family history, these hereditary amyloidoses are initially misdiagnosed as acquired amyloidoses and mistreated similarly with chemotherapy [4–6].

Serum protein electrophoresis (SPEP) and urine protein electrophoresis (UPEP) with immunofixation are part of the initial workup but are neither sensitive nor specific for amyloidosis [1–12]. The presence of paraprotein in serum and urine can actually be misleading because subtle gammopathies can be present even in the general population [4]. Our patient also had a small degree of kappa monoclonal gammopathy on serum and kappa monotypic plasma cells in the bone marrow, raising suspicion for AL amyloidosis. Biopsy of the involved organ is usually the initial step for reaching the definite diagnosis of amyloidosis [1–5]. The next step after the diagnosis of amyloidosis is the identification of the protein type. Because all of the different types of amyloid fibrils appear morphologically similar and cannot be differentiated on light or electron microscopy, additional testing is required [13]. Immunofluorescence or immunohistochemical staining of tissue uses antibodies directed against known amyloidogenic proteins to identify them and are routinely used in the clinical setting [4]. However, the most direct method of identification is mass spectrometry or amino acid sequencing of proteins which are extracted from amyloid deposits [5]. The diagnosis of apolipoprotein C-II associated with renal amyloidosis in our patient was made by laser microdissection (LMD) and liquid chromatography mass spectrometry (LCMS) performed on peptides extracted from Congo red positive dissected areas.

Apolipoprotein C-II is an 8914 Da exchangeable component of VLDL and chylomicrons and acts as a cofactor for lipoprotein lipase and therefore has a role in cholesterol transport [7]. In the presence of polar lipid (phospholipids), it has an *α* helical structure [14–17]. However, in a lipid-free environment, it adopts a *β* pleated structure and can self-aggregate to form twisted ribbon-like homogenous amyloid fibrils [6, 14–18]. The mechanism of amyloid fibril formation from apolipoprotein C-II has been studied in vitro in many studies [7, 14, 15, 18–20]; it is thought that the fibril formation is due to a gene mutation associated with apolipoprotein C-II [18].

Based on the initial bone marrow and renal biopsy findings, our patient was initially treated for AL amyloidosis with chemotherapy, which has no role and can be harmful in hereditary amyloidosis in general, including apolipoprotein C-II-associated disease [4–6]. Correct identification of the amyloid protein as apolipoprotein C-II was imperative in stopping the chemotherapy in the case presented. Since the number of amyloidogenic proteins being identified is increasing, making a correct and definitive diagnosis is difficult [9]. At the time of initial diagnosis of apolipoprotein C-II in this patient, a series of 8 patients with apolipoprotein C-II amyloid deposits in renal biopsy from the Mayo clinic was not published [21].

LMD/MS is a relatively new technique used for diagnosis and typing of amyloidosis [22]. This technique includes laser microdissection of the glomeruli under microscope to separate the focus of interest from rest of the tissue [23, 24]. This can be performed on paraffin block without any special tissue requirements and even older biopsy specimens can be used [23, 24]. Microdissected fragments are digested into peptides overnight and subsequently analyzed by liquid chromatography electrospray tandem mass spectrometry (MS) [23, 24]. MS raw data files are queried using various algorithms to generate protein profile. The MS data show spectra that match a particular protein based on the amino acid sequence available in the database [23]. Unique peptides and spectra are distinctive to the particular protein [23]. As a list of protein constituents of the amyloid is generated, all known amyloid types can be theoretically identified by this technique [12]. A higher mass spectra value also indicates a higher confidence in the protein identification [23]. LMD/MS has a special role in identification of amyloid protein especially when a definitive diagnosis cannot be reached with immunofluorescence that is common in hereditary amyloidosis [8, 22, 23]. However, this technique is not readily and widely available, leading to a delay in diagnosis [23].

LMD/MS is an emerging technique that shows great promise for the diagnosis and understanding of kidney diseases including amyloidosis [23] and was crucial in the identification of apolipoprotein C-II causing amyloidosis in our case. Further genetic testing with gene sequencing should also be done to confirm the gene mutation after identification of the amyloid protein [6].

## 4. Conclusion

Apolipoprotein C-II associated familial amyloidosis is a rare form of systemic amyloidosis affecting the kidneys. Early accurate diagnosis of this disease is important to avoid unnecessary cost and side effects associated with inappropriate treatment as well as assess prognosis and provide appropriate genetic counseling. Research studies for these hereditary amyloid proteins are necessary for determining the prevalence of these rare under diagnosed familial systemic amyloidoses. This is very important in the future development of treatment for the now incurable condition. Technologies such as laser microscopy and mass spectrometry have been valuable in the appropriate diagnosis of patients with hereditary amyloidosis and should be routinely used in clinical practice when routine assessment fails to reach an accurate diagnosis.

## Figures and Tables

**Table 1:** Laboratory values.

	Values	Reference range
White blood count (WBC)	4.9 billions/L	3.3–10.7 billions/L
Hemoglobin	12.4 g/dL	12.1–15 g/dL
Platelet	190 billions/L	150–400 billions/L
Sodium	141 mmol/L	135–145 mmol/L
Potassium	4.8 mmol/L	3.5–5.2 mmol/L
Blood urea nitrogen (BUN)	42 mg/dL	8–22 mg/dL
Creatinine	2.3 mg/dL	0.60–1.4 mg/dL
Phosphorus	5.6 mg/dL	2.3–4.3 mg/dL
Chloride	116 mmol/L	98–110 mmol/L
Glucose	109 mg/dL	60–99 mg/dL
Calcium	8.1 mg/dL	8.5–10.5 mg/dL
Alkaline phosphatase	63 U/L	30–110 U/L
Aspartate aminotransferase	22 U/L	10–37 U/L
Alanine aminotransferase	19 U/L	8–37 U/L
Albumin	3.2 mg/dL	3.5–4.9 mg/dL
Vitamin D (25-hydroxy)	7 ng/mL	25–80 ng/mL
Parathyroid hormone (PTH)	82 pg/mL	9–69 pg/mL
Urine protein/creatinine ratio	6.3 mg/g	<0.2 mg/g
Complement C3	91 mg/dL	70–176 mg/dL
Complement C4	18.9 mg/dL	12.1–42.9 mg/dL
Creatine Kinase (CK)	134 U/L	30–150 U/L
Immunoglobulin G (IgG)	164 mg/dL	564–1765 mg/dL
Immunoglobulin A (IgA)	20 mg/dL	85–385 mg/dL
Immunoglobulin M (IgM)	24 mg/dL	45–250 mg/dL

## References

[1] Seldin D. C., Skinner M., Longo D. L., Longo D. L. (2012). Amyloidosis. *Harrison's Principles of Internal Medicine*.

[2] Buxbaum J. N., Tagoe C. E. (2000). The genetics of the amyloidoses. *Annual Review of Medicine*.

[3] Kyle R. A., Linos A., Beard C. M. (1992). Incidence and natural history of primary systemic amyloidosis in Olmsted County, Minnesota, 1950 through 1989. *Blood*.

[4] Lachmann H. J., Booth D. R., Booth S. E. (2002). Misdiagnosis of hereditary amyloidosis as AL (primary) amyloidosis. *The New England Journal of Medicine*.

[5] Dember L. M. (2006). Amyloidosis-associated kidney disease. *Journal of the American Society of Nephrology*.

[6] Saraiva M. J. (2002). Sporadic cases of hereditary systemic amyloidosis. *The New England Journal of Medicine*.

[7] Ryan T. M., Griffin M. D. W., Bailey M. F., Schuck P., Howlett G. J. (2011). NBD-labeled phospholipid accelerates apolipoprotein C-II amyloid fibril formation but is not incorporated into mature fibrils. *Biochemistry*.

[8] Sethi S., Theis J. D., Leung N. (2010). Mass spectrometry-based proteomic diagnosis of renal immunoglobulin heavy chain amyloidosis. *Clinical Journal of the American Society of Nephrology*.

[9] Merlini G., Bellotti V. (2003). Molecular mechanisms of amyloidosis. *The New England Journal of Medicine*.

[10] Sipe J. D., Cohen A. S. (2000). Review: history of the amyloid fibril. *Journal of Structural Biology*.

[11] Dember L. M., Shephard J. O., Nesta F., Stone J. R. (2005). Case 15-2005: an 80-year-old man with shortness of breath, edema, and proteinuria. *The New England Journal of Medicine*.

[12] Vrana J. A., Gamez J. D., Madden B. J., Theis J. D., Bergen H. R., Dogan A. (2009). Classification of amyloidosis by laser microdissection and mass spectrometry-based proteomic analysis in clinical biopsy specimens. *Blood*.

[13] Sunde M., Blake C. C. F. (1998). From the globular to the fibrous state: protein structure and structural conversion in amyloid formation. *Quarterly Reviews of Biophysics*.

[14] MacRaild C. A., Hatters D. M., Howlett G. J., Gooley P. R. (2001). NMR structure of human apolipoprotein C-II in the presence of sodium dodecyl sulfate. *Biochemistry*.

[15] MacRaild C. A., Howlett G. J., Gooley P. R. (2004). The structure and interactions of human apolipoprotein C-II in dodecyl phosphocholine. *Biochemistry*.

[16] Tajima S., Yokoyama S., Kawai Y., Yamamoto A. (1982). Behavior of apolipoprotein C-II in an aqueous solution. *The Journal of Biochemistry*.

[17] Hatters D. M., Lawrence L. J., Howlett G. J. (2001). Sub-micellar phospholipid accelerates amyloid formation by apolipoprotein C-II. *FEBS Letters*.

[18] Hatters D. M., MacPhee C. E., Lawrence L. J., Sawyer W. H., Hewlett G. J. (2000). Human apolipoprotein C-II forms twisted amyloid ribbons and closed loops. *Biochemistry*.

[19] Wilson L. M., Pham C. L. L., Jenkins A. J. (2006). High density lipoproteins bind A*β* and apolipoprotein C-II amyloid fibrils. *Journal of Lipid Research*.

[20] Hatters D. M., Minton A. P., Howlett G. J. (2002). Macromolecular crowding accelerates amyloid formation by human apolipoprotein C-II. *The Journal of Biological Chemistry*.

[21] Nasr S. H., Dasari S., Hasadsri L. (2016). Novel type of renal amyloidosis derived from apolipoprotein-CII. *Journal of the American Society of Nephrology*.

[22] Sethi S., Vrana J. A., Theis J. D. (2012). Laser microdissection and mass spectrometry-based proteomics aids the diagnosis and typing of renal amyloidosis. *Kidney International*.

[23] Sethi S., Vrana J. A., Theis J. D., Dogan A. (2013). Mass spectrometry based proteomics in the diagnosis of kidney disease. *Current Opinion in Nephrology & Hypertension*.

[24] Jain D., Green J. A., Theis J. D., Sethi S. (2014). Membranoproliferative glomerulonephritis: the role for laser microdissection and mass spectrometry. *American Journal of Kidney Diseases*.

